# [Corrigendum] Changes of type II collagenase biomarkers on IL‑1β‑induced rat articular chondrocytes

**DOI:** 10.3892/etm.2023.12040

**Published:** 2023-05-23

**Authors:** Xiangying Ma, Zhiheng Zhang, Meilun Shen, Yuanqiang Ma, Rouqian Li, Xiaodi Jin, Li Gao, Zhi Wang

Exp Ther Med 21:582, 2021; DOI: 10.3892/etm.2021.10014

Following the publication of the above article, an interested reader drew to the authors’ attention that, for the morphological observations of chondrocytes portrayed in Fig. 1 on p. 3, the image selected to show the primary cultures (Fig. 1A) and the chondrocytes of passage 4 (Fig. 1D) appeared to be the same. The authors have re-examined their original data, and realize that this image was selected incorrectly for Fig. 1D. The corrected version of Fig. 1, showing the correct data for the chondrocytes of passage 4, is shown below. Note that the error made in assembling this figure incorrectly did not affect the overall conclusions reported in the paper, and all the authors agree with the publication of this corrigendum. The authors are grateful to the Editor of *Experimental and Therapeutic Medicine* for allowing them the opportunity to publish this. They also apologize to the readership for any inconvenience caused.

## Figures and Tables

**Figure 1:**
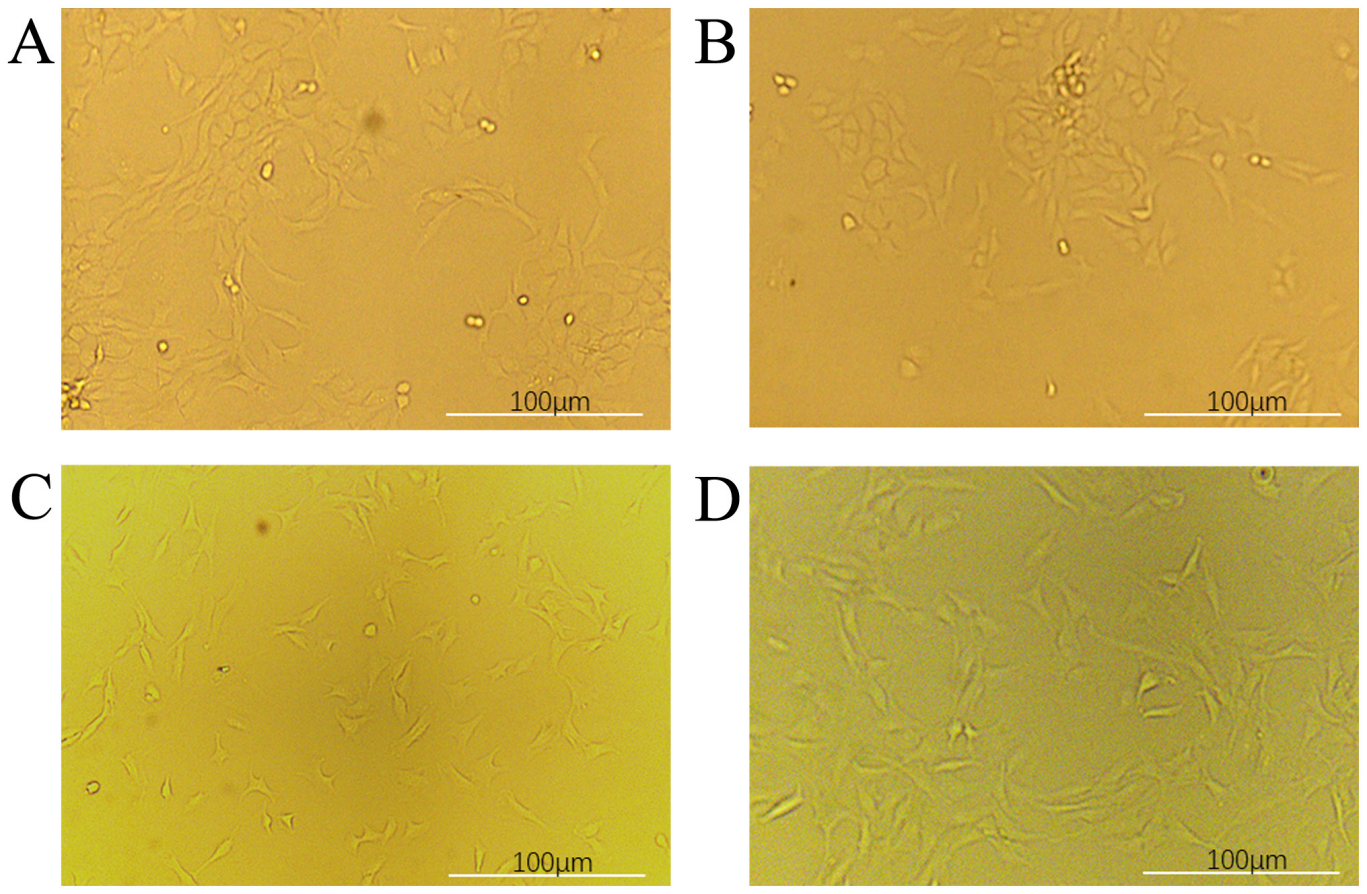
Morphological observation of chondrocytes (x100). (A) Primary cultures. (B) Chondrocytes of passage 2. (C) Chondrocytes of passage 3. (D) Chondrocytes of passage 4.

